# Supplementary material for: Low-cost sample preservation methods for high-throughput processing of rumen microbiomes
Source: Anim Microbiome. 2022 Jun 6;4:39. doi: 10.1186/s42523-022-00190-z (PMC9171989; doi:10.1186/s42523-022-00190-z)
Supplement: Supplementary file 3 — Additional file3. Supplementary figures for visual assessment of sheep rumen samples using different preservation methods. Fig. S2 8 mL vial containing the sheep rumen sample preserved with TNx2, GHx2 or EtOH. Fig. S3 2 mL vials containing TNx2, GHx2 or EtOH and sheep rumen sample after the “bead-beating” step. Fig. S4 2 mL vials containing sheep rumen samples preserved using TNx2, GHx2, EtOH and the GRC method after centrifugation. Fig. S5) Principal component analysis (PCA) of the log10 relative abundance matrix using the RB approach for all non-failed samples (> 100,000 reads) including positive control samples. [file 42523_2022_190_MOESM3_ESM.docx]

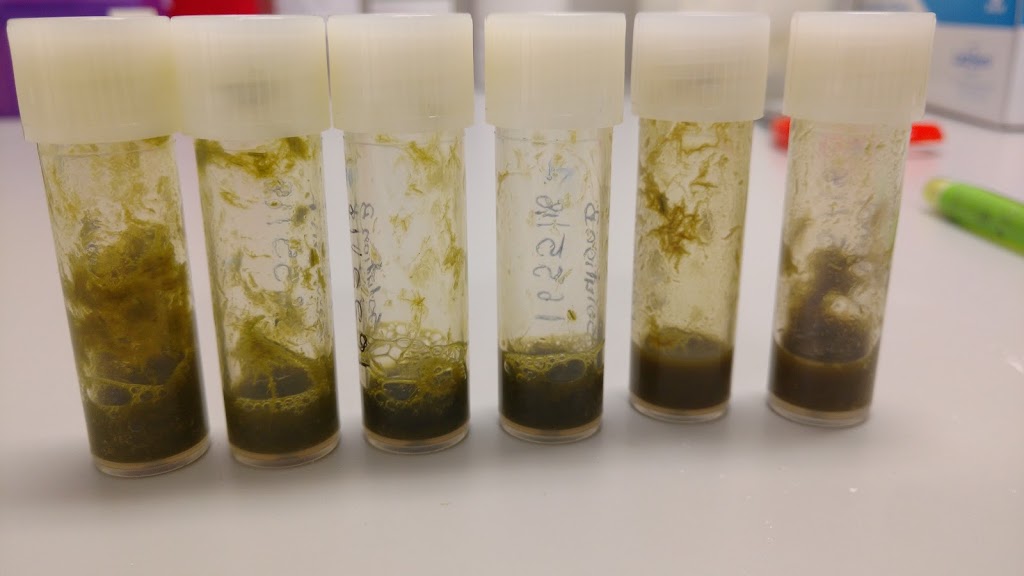


**(a)**

**(b)**

**(c)**

**(b)**

**(c)**

**(a)**

**Fig. S2** 8 mL vials containing the same sheep rumen sample preserved with (a) TNx2, (b) GHx2 or (c) EtOH.

**(a)**

**(c)**

**(b)**


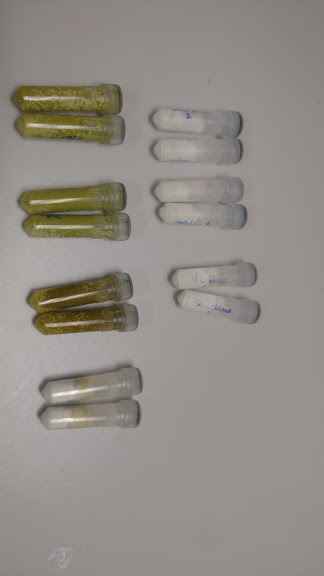


**Fig. S3** 2 mL vials containing (a) TNx2, (b) GHx2, or (c) EtOH with the same sheep rumen sample after the “bead-beating” step.

| **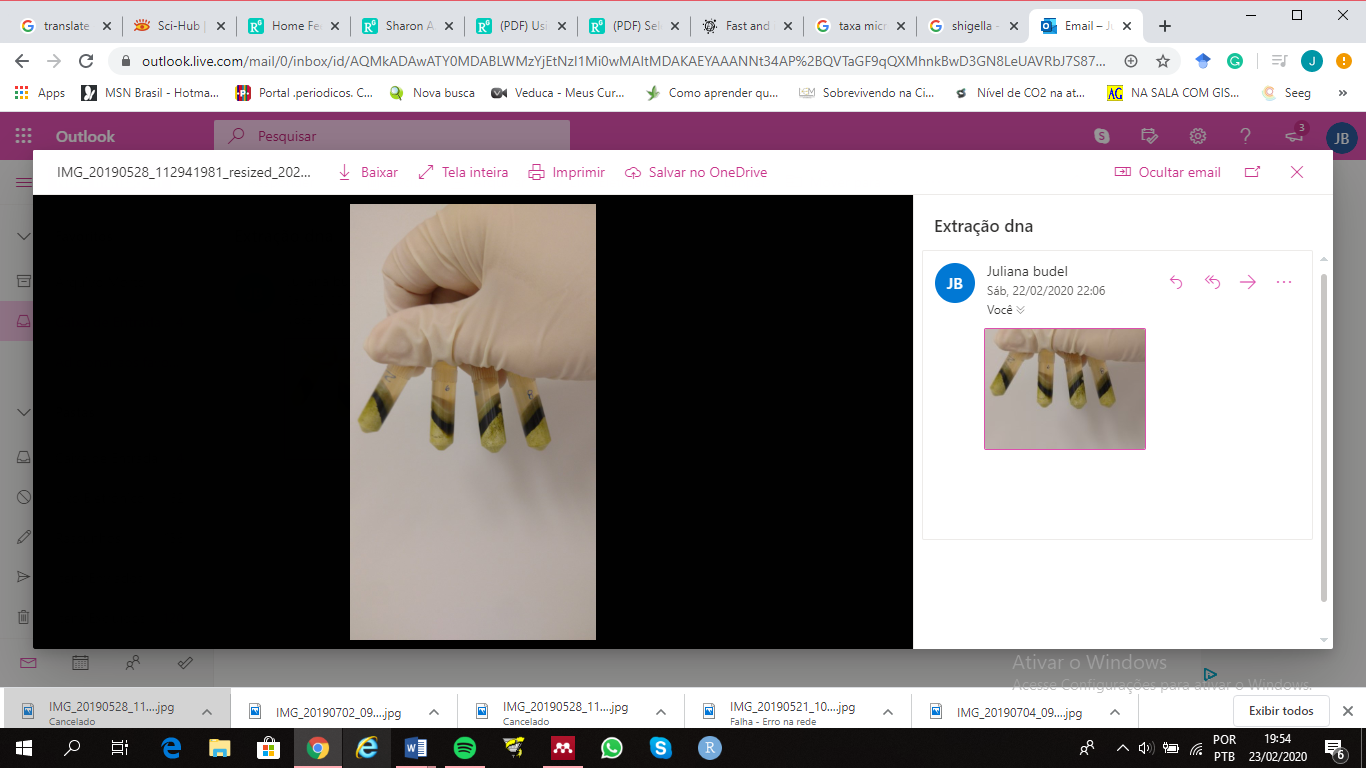** | 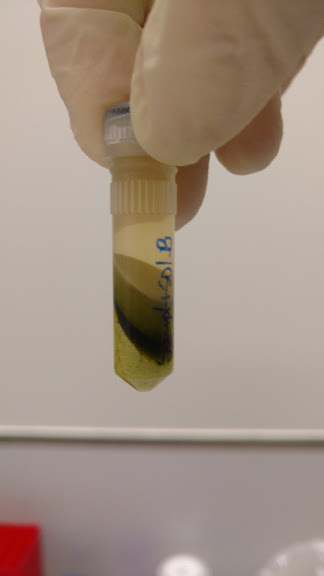 | **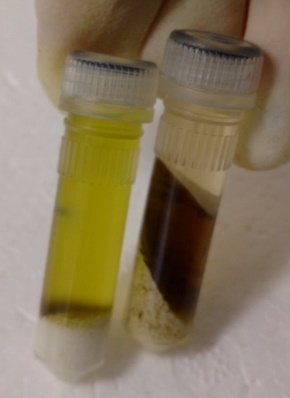** | **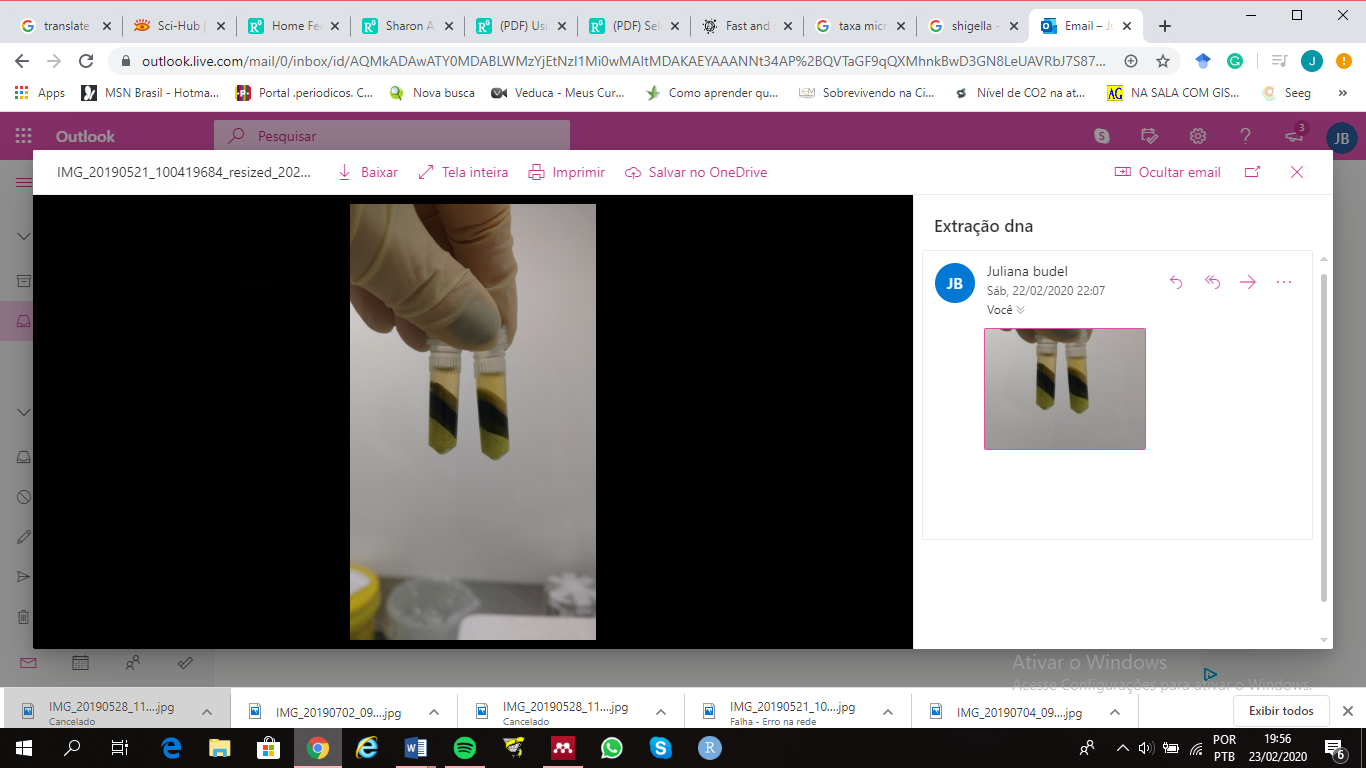** |
| --- | --- | --- | --- |
| **(a)** | **(b)** | **(c)** | **(d)** |

**Fig. S4** 2 mL vials containing the same sheep rumen samples preserved using (a) TNx2, (b) GHx2, (c) EtOH, and (d) the GRC method after centrifugation.


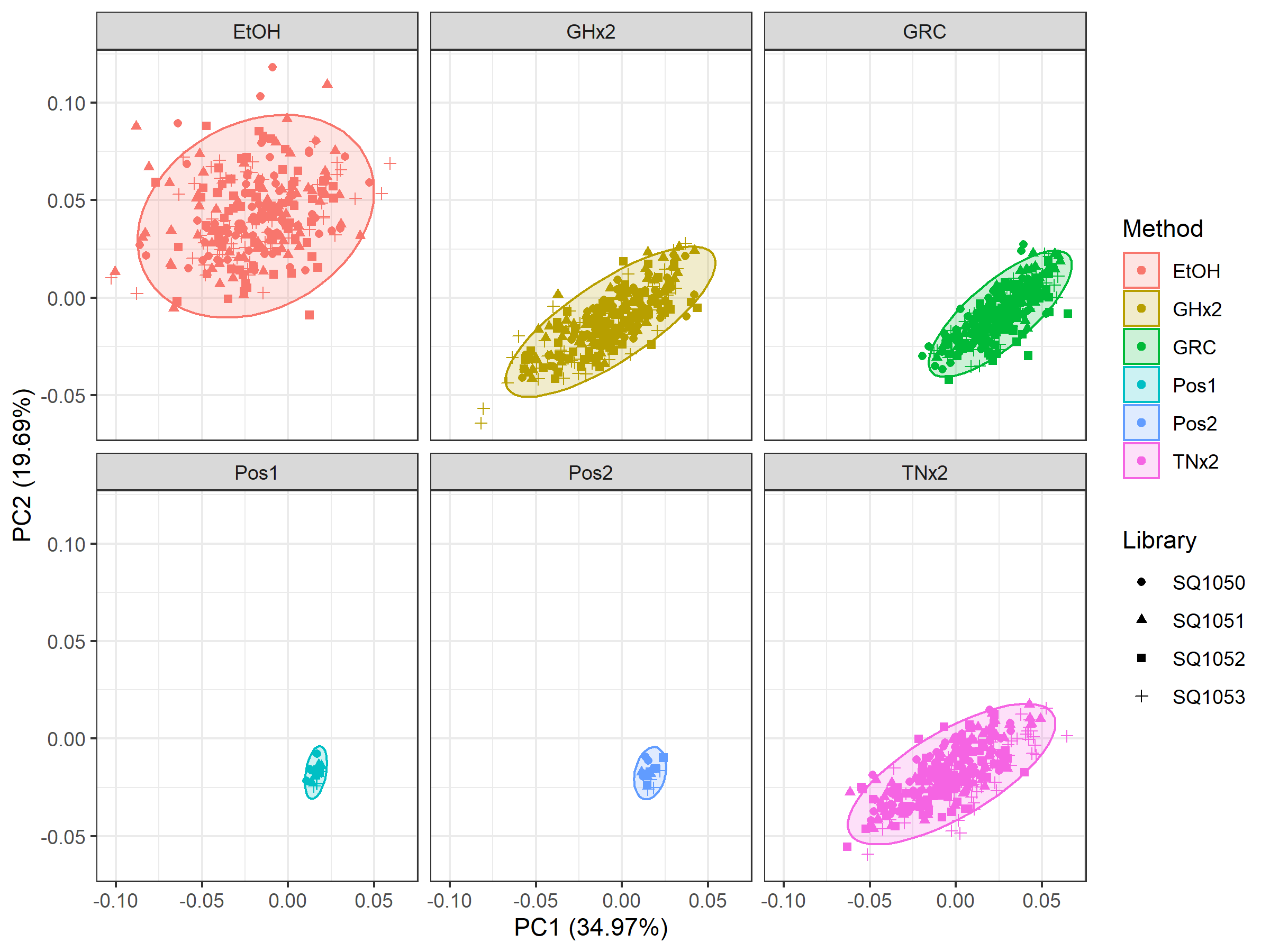


**Fig. S5** Principal component analysis (PCA) of the log_10_ relative abundance matrix using the RB approach for all non-failed samples (>100k reads) including positive control samples. The 32 positive controls samples were split equally across two individuals (labelled Pos1 and Pos2) and were preserved using the GRC method.
